# Case Report: Infliximab for hydroxychloroquine-induced AGEP with IL17A and IL36RN mutations: a case report and management considerations

**DOI:** 10.3389/fphar.2025.1580170

**Published:** 2025-07-21

**Authors:** Xiaoyun Jiang, Chunting Hua, Chenxi Feng, Hao Cheng

**Affiliations:** Department of Dermatology, Sir Run Run Shaw Hospital, School of Medicine, Zhejiang University, Hangzhou, China

**Keywords:** infliximab, hydroxychloroquine, AGEP, IL17A, IL36RN, pregnant

## Abstract

Acute generalized exanthematous pustulosis (AGEP) is a severe cutaneous drug reaction that typically resolves rapidly following withdrawal of the offending drug and administration of systemic corticosteroids. Infliximab, a biologic agent that inhibits TNF-α, is widely used in the treatment of various inflammatory diseases. Individuals with specific genetic risk factors may be more susceptible to severe drug-induced cutaneous reactions. Here, we report the first case of successful infliximab treatment in a pregnant woman with IL17A and IL36RN mutations who developed severe AGEP after 24 days of hydroxychloroquine use and failed to respond to conventional therapy.

## Introduction

IL-17A can induce the expression of IL-36γ in human dermal microvascular endothelial cells and they were demonstrated to have synergistic activity with TNF-α by inducing the release of inflammatory cytokines/chemokines (Mercurio et al., 2020). Approximately a decade ago, researchers proposed that due to IL36RN mutations, the IL-36 signaling becomes uncontrolled, which may lead to the occurrence of AGEP (Nakai et al., 2015). However, further validation was limited by the scarcity of clinical data. Some scholars have conducted a retrospective study on female patients diagnosed with hydroxychloroquine-induced AGEP. They found that the average time from the onset of hydroxychloroquine to the appearance of symptoms is 40 days and IL36RN is not consistently present (Chaabouni et al., 2021). Recently, a study revealed a significant association between IL36RN gene variants and AGEP by conducting Sanger sequencing on six genes in a cohort of 126 patients with sterile pustulosis (Chen et al., 2024). The elevated expression level of IL-17A was also observed in AGEP (Kakeda et al., 2014), and administration of IL-17A inhibitor showed rapid clearance of AGEP (Wen et al., 2024). But the underlying mechanisms of have not been completely elucidated.

Infliximab is a monoclonal antibody targeting tumor necrosis factor-alpha. Current evidence suggests that infliximab is safe for use during pregnancy and the first 2 months of gestation (Djokanovic et al., 2011) and does not seem to exhibit any heightened risk of adverse pregnancy outcomes (Kane, 2018). Study found that the drug concentration of infliximab in women with inflammatory bowel disease was still detectable in their infants up to 6 months after birth, indicating that infliximab can be transferred across the placenta (Julsgaard et al., 2016).

## Case description

A 26-year-old woman presented to our hospital with infertility. The value of 25(OH)D was as low as 6.62 μg/mL, without an abnormal thyroid profile or antibody. B-ultrasonography of the uterine artery indicated the disappearance of the left diastolic blood flow spectrum. A high risk of methylenetetrahydrofolate reductase (MTHFR) deficiency was also considered. She then began taking vitamin D 5000 iu, calcitriol 0.5 μg, hydroxychloroquine (200 mg), and prednisone (5 mg daily). After 2 weeks, the embryos were successfully transplanted. However, the patient developed edematous erythema on the arm and cheek, without obvious pruritus, 3 days after embryo transplantation. Then erythema gradually appeared throughout the body with tiny pustules in the next 5 days, and she had fever with the highest body temperature of 38.9°C in the meantime.

**FIGURE 1:**
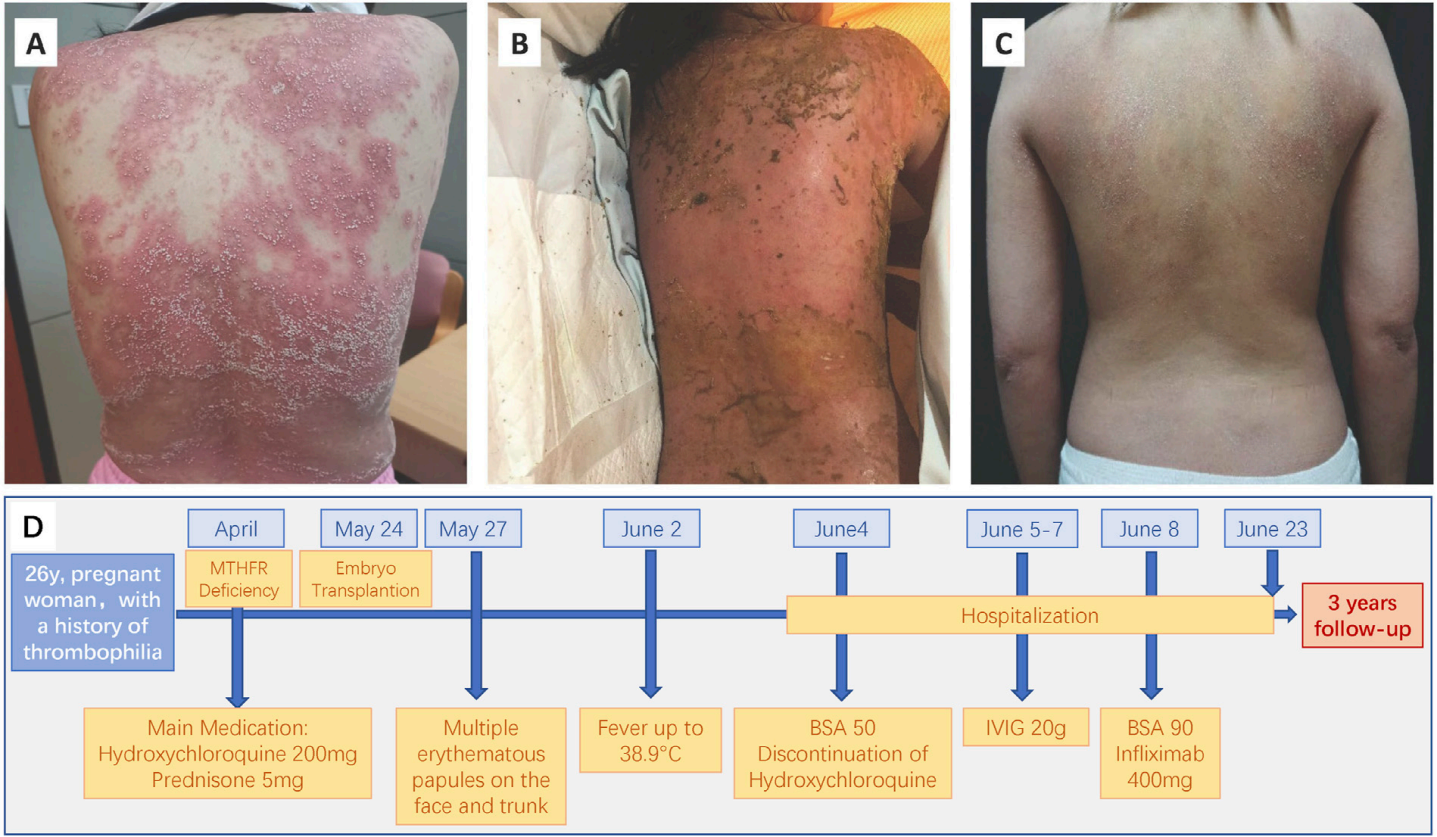
Clinical photographs of the patient. Before **(A)**, 4 days after **(B)**, and 2 weeks after **(C)** infliximab treatment. **(D)** Time line.

After receiving hydroxychloroquine for 24 days, she visited the hospital for medical help. No mucosal lesions were observed but the skin lesions had already reached 50% of the body surface area (BSA) (Figure 1A). The white blood cell count and blood neutrophil percentage were elevated to 27.1 × 10^9^/L and 93.3%, respectively. Together with the high levels of the C reaction protein (155.6 mg/L) and D dimer (5.62 μg/mL). Hydroxychloroquine was discontinued immediately, and intravenous immunoglobulin (20 g) was administered daily for 3 days. However, the patient gradually presented with toxic epidermal necrolysis (TEN) with no mucosal involvement. She had diffuse exfoliation and pustules merged into bullae with skin lesions of over 90% BSA. The patient refused systemic glucocorticoids to avoid adverse fetal effects.

Furthermore, HLA typing revealed that the patient had three HLA alleles (A*02:07, B*46:01, DRB1*04:03), which are associated with other drug reactions, and eight alleles (A*26:01, B*27:05, C*01:02, C*02:02, DRB*104:01, DQB*103:01, DQB*03:02, DPB*105:01.05:01), which have not yet been reported. Mutations in IL17A and IL36RN were also identified (Table 1).

**TABLE 1:** Mutations of the patient.

Target	Location	Nucleotide	ClinVar variation ID
IL36RN	chr2:113818520	NM_012275.3:c.115 + 6T>C	40005
	chr2:113816942	NM_012275.3:c.-27-47A>C	2628159
	chr2:113819614	NM_012275.3:c.116-87T>C	2688401
IL17A	chr6:52051274	NM_002190.3:c.27 + 18G>A	1273159

Though Infliximab was reported to be safe and effective in a 24-year-old pregnant woman with generalized pustular psoriasis (Babuna and Polat, 2020), there is still limited research on the safety of infliximab use in pregnant patients. Following an assessment by a multidisciplinary team, which included dermatologists and a rheumatologist specializing in pregnancy immunology, the patient expressed acceptance of potential risks, including adverse effects on the fetus and unknown long-term consequences and received an intravenous injection of infliximab (400 mg) on the 15th day post-embryo transfer. The skin exudation decreased spontaneously, and the pustules resolved with desquamation (Figure 1B). After 2 weeks of infliximab treatment, generalized rash has largely subsided, and the patient has been discharged (Figure 1C). During the subsequent 3-year follow-up, the patient experienced no recurrence of drug rash and no adverse events occurred. Preparations are currently underway for the next embryo transplantation.

## Discussion

AGEP typically presents with an acute onset. It features small, non - follicular pustules on edematous erythema, accompanied by fever. Patients usually have a relevant drug - taking history. Novo pustular psoriasis usually has a more gradual onset. It shows erythema with non - infectious pustules. Patients may have a personal or family history of psoriasis. Neutrophilic pustular eruption can be linked to various underlying conditions or medications. It presents with pustules and red plaques. Its onset may be associated with systemic diseases or drug use.

In our case, the patient presented with a sudden onset of widespread, non-follicular pustules and erythema after the initiation of hydroxychloroquine, which is highly suggestive of AGEP. The patient’s Naranjo score was 5, indicating a high probability of a drug-induced reaction. Additionally, the absence of a prior history of psoriasis and the lack of involvement of typical psoriasis sites (e.g., scalp, elbows, knees) further supports AGEP over *de novo* pustular psoriasis. In terms of neutrophilic pustular eruptions, these can be associated with various conditions, including infections, malignancies, or inflammatory diseases. However, the temporal association with medication use and the clinical presentation of AGEP were more consistent with a drug-induced reaction.

In the management of severe cutaneous adverse reactions such as acute generalized exanthematous pustulosis (AGEP), particularly during pregnancy, selecting the appropriate treatment is of utmost importance. When genetic mutation assessment is not feasible, corticosteroids remain our first-line consideration due to their well-established efficacy and safety profile in pregnancy. The multidisciplinary team (MDT) plays a pivotal role in ensuring that all aspects of the patient’s condition are comprehensively evaluated. The decision to use any biologic agent during pregnancy must be approached with caution, given the limited data on their safety in pregnant women. While biologics offer targeted and effective treatment options for severe dermatological conditions, their use in pregnancy is often restricted due to the potential risks to the developing fetus. Ensuring that the patient is well-informed about the potential benefits and risks involved is essential for making shared decisions.

In most cases, AGEP has a rapid onset within hours or days of drug exposure and resolves spontaneously within 1–2 weeks after drug withdrawal or systemic therapy (Parisi et al., 2023). Few cases develop TEN-like AGEP (van Hattem et al., 2014), identifying danger signs helps in immediate and appropriate treatment. Mutations in IL36RN have been considered relevant to the high risk of AGEP development (Hadavand et al., 2022) and high percentages of Th17 cells have been observed in AGEP patients (Kabashima et al., 2011).

There are ethical concerns regarding the investigation of the use of biologic agents in pregnant and lactating patients. To date, another case report has documented the use of infliximab in a pregnant woman with systemic lupus erythematosus who developed hydroxychloroquine induced AGEP but underwent caesarean section after 1 month for the risk of infection (Tsurane et al., 2023). Whether the patient has any specific genetic defects remains unknown (Supplementary Table S1). To fully understand the impact of infliximab on the development and maturation of the immune system in pregnant women and fetuses, long-term follow-up results from prospective studies are needed.

In our study, intravenously injection of infliximab was used on the 15th day post-embryo transfer. It is worth noting that regular monitoring of HCG levels revealed that the HCG level was 200 IU/L on the 12th day, 639 IU/L on the 15th day, and 1185 IU/L on the 23rd day post-embryo transfer, indicating poor embryonic development. Consequently, a medical abortion was performed on the 24th day post-embryo transfer. It is currently impossible to confirm whether the treatment affects embryonic development.

The presence of genetic mutations may affect the latency and resolution time of hydroxychloroquine -induced AGEP. The functional impact of IL-17A mutations in AGEP requires further investigation. Here, we report an IL17A mutation with no functional evidence and three IL36RN mutations, hoping to provide corroborating evidence for future mechanistic studies and clinical medication decision-making.

## Conclusion

Hydroxychloroquine should be administered with caution to patients with mutations in the IL17A and IL36RN. Moreover, there are no relevant reports on genetic risk factors for the development of hydroxychloroquine-induced drug reactions. Patients with a history of drug reactions should receive personalized medicine based on their genetic patterns.

## Data Availability

The datasets presented in this article are not readily available because of ethical and privacy restrictions. Requests to access the datasets should be directed to the corresponding author.
