# Supplementary material for: Case Report: Infliximab for hydroxychloroquine-induced AGEP with IL17A and IL36RN mutations: a case report and management considerations
Source: Front Pharmacol. 2025 Jul 21;16:1580170. doi: 10.3389/fphar.2025.1580170 (PMC12319033; doi:10.3389/fphar.2025.1580170)
Supplement: Supplementary file 1 [file Supplementaryfile1.docx]

| **PMID** | **Clinical Features** | **Type** | **Latency** | **Treatment** | **Outcome** | **Mutation** |
| --- | --- | --- | --- | --- | --- | --- |
| 20548896 | 38y，female | AGEP | 3 weeks | Drug withdrawal + corticosteroids | Improved | NA |
| 19727917 | 67y,female | AGEP/TEN | 3 weeks | Drug withdrawal+ corticosteroids+ivig | Improved | NA |
| 11961185 | 65y,female | SJS | 2 weeks | Drug withdrawal + corticosteroids | Improved | NA |
| 24138979 | 48y，female | AGEP | 2 weeks | Drug withdrawal + corticosteroids | Improved | NA |
| 31067554 | 9y,femal | AGEP | 1 month | Drug withdrawal + supportive care | Improved | NA |
| 26273160 | 67y,female | AGEP | 15 days | Drug withdrawal + cyclosporine | Improved | NA |
| 38013380 | 28y，female | AGEP | 5 days | Drug withdrawal + corticosteroids | Improved | CARD14 |
| 36420890 | 30y,female,pregnant | AGEP | 7 weeks | Drug withdrawal+Infliximab | Improved | NA |
| Our case | 28y,female,pregnant | AGEP | 24 days | Drug withdrawal+ivig+Infliximab | Improved | IL17A and IL36RN |

Supplementary Table 1. Comparison with other cases.
